# Lipid Nanoparticle Delivery Systems to Enable mRNA-Based Therapeutics

**DOI:** 10.3390/pharmaceutics14020398

**Published:** 2022-02-11

**Authors:** Sean C. Semple, Robert Leone, Christopher J. Barbosa, Ying K. Tam, Paulo J. C. Lin

**Affiliations:** Acuitas Therapeutics, 6190 Agronomy Road, Vancouver, BC V6T 1Z3, Canada; ssemple@acuitastx.com (S.C.S.); rleone@acuitastx.com (R.L.); cbarbosa@acuitastx.com (C.J.B.); ytam@acuitastx.com (Y.K.T.)

**Keywords:** lipid nanoparticles, siRNA-LNP, mRNA-LNP, prophylactic vaccines, gene editing, mRNA-based therapeutics

## Abstract

The world raced to develop vaccines to protect against the rapid spread of SARS-CoV-2 infection upon the recognition of COVID-19 as a global pandemic. A broad spectrum of candidates was evaluated, with mRNA-based vaccines emerging as leaders due to how quickly they were available for emergency use while providing a high level of efficacy. As a modular technology, the mRNA-based vaccines benefitted from decades of advancements in both mRNA and delivery technology prior to the current global pandemic. The fundamental lessons of the utility of mRNA as a therapeutic were pioneered by Dr. Katalin Kariko and her colleagues, perhaps most notably in collaboration with Drew Weissman at University of Pennsylvania, and this foundational work paved the way for the development of the first ever mRNA-based therapeutic authorized for human use, COMIRNATY^®^. In this Special Issue of Pharmaceutics, we will be honoring Dr. Kariko for her great contributions to the mRNA technology to treat diseases with unmet needs. In this review article, we will focus on the delivery platform, the lipid nanoparticle (LNP) carrier, which allowed the potential of mRNA therapeutics to be realized. Similar to the mRNA technology, the development of LNP systems has been ongoing for decades before culminating in the success of the first clinically approved siRNA-LNP product, ONPATTRO^®^, a treatment for an otherwise fatal genetic disease called transthyretin amyloidosis. Lessons learned from the siRNA-LNP experience enabled the translation into the mRNA platform with the eventual authorization and approval of the mRNA-LNP vaccines against COVID-19. This marks the beginning of mRNA-LNP as a pharmaceutical option to treat genetic diseases.

## 1. Introduction

The world has forever changed ever since the first reported case of COVID-19 infection in Wuhan China in the end of 2019. As of November 2021, the World Health Organization (WHO) has reported over 258 million cases with over 5.1 million deaths worldwide. The health care systems of many countries have been challenged beyond their limits, the global economy has been impacted significantly due to the restrictions placed to curb the infection rate and psychosocial impact will manifest for many years to come [1,2]. However, one positive outcome from this adversity was the manner in which the scientific community, both academic and industry, came together in a concerted effort to develop safe and effective vaccines to protect humanity. The various collaborations resulted in 159 vaccine candidates and 515 clinical trials evaluating a variety of modalities (https://covid19.trackvaccines.org as of 25 November 2021) ranging from the traditional protein-based and inactivated viral vaccines to the recombinant viral based-vaccines to the mRNA-LNP vaccines, to name a few. Over the course of the pandemic, several promising vaccines of different modalities reached Phase 3 clinical studies with just a smaller subset currently authorized for emergency use. From the handful of vaccines granted emergency use worldwide, the two vaccines that were most successful during the pandemic were the mRNA-based vaccines. The speed of creating these vaccines from the first reported case to the sequencing of the SARS-CoV-2, through completion of the full course of clinical trials to regulatory authorization for emergency use within 1 year was unprecedented and provides a vaccine development framework for future pandemics. The vaccine development was not only fast, but also highly effective. mRNA-based vaccines were proven to be safe with >94% effectiveness with Phase 3 studies consisting of 43,448 participants sponsored by BioNTech/Pfizer [3] and 30,420 participants by Moderna [4]. Importantly, the efficacy of these vaccines is still being monitored and continues to demonstrate protection over time and across new mutational variants [5,6,7,8,9].

In addition, the manufacturing of BNT162b2 (BioNTech/Pfizer’s COMIRNATY^®^) and mRNA-1273 (Moderna’s Spikevax^®^ COVID-19 Vaccine) were particularly impressive considering how quickly both groups have delivered billions of doses to address the global demands to provide protection against serious illness, hospitalization, and death due to SARS-CoV-2 infection. This can be attributed to a very well planned and coordinated effort by all the groups involved during development. However, this could only be achieved by the strong foundation established by decades of ground-breaking work by those who envisioned mRNA as a therapeutic. mRNA was first reported by Francois Jacob, Sydney Brenner, and Jim Watson in 1961 [10]. Since then, it is well established that the main function of mRNA in life is to serve as the bridge between the coding sequence of DNA to the production of functional protein. In nature, since mRNA is labile and short lived and due to its sensitivity to enzymatic degradation, it was extremely difficult to harness its potential as a therapeutic. It was the persistence of several mRNA experts that turned the concept into reality. One of the most productive researchers in the mRNA field who contributed immensely to the understanding and design of synthetic mRNA is Dr. Katalin Kariko. The fundamental studies pioneered by Dr. Kariko, her colleague Dr. Drew Weissman, and many others in the mRNA field showed the importance of the UTR selection, codon optimization, nucleoside modification, purification, capping technologies, and length of poly A tails in the stability and translatability of the mRNA while reducing immune stimulation [11,12,13,14,15,16,17,18,19,20,21,22,23,24,25,26,27]. Over the years, Acuitas Therapeutics had the pleasure to collaborate in multiple successful projects with Drs. Kariko and Weissman [28,29,30,31,32,33] and is honored to contribute in this Special Issue of Pharmaceutics dedicated to Dr. Kariko. 

The understanding of mRNA and how it can be harnessed as a therapeutic has advanced significantly over the decades; however, mRNA is still vulnerable to digestion by circulating nucleases, is rapidly cleared, and has very poor intracellular uptake properties due to its sheer size and charge. Although naked mRNA can still be taken up by cells, this process remains very inefficient [34]. In order to protect and facilitate intracellular delivery of mRNA, an effective and safe delivery system was essential. Both of the mRNA-based vaccines developed by BioNTech/Pfizer and Moderna rely on a lipid nanoparticle (LNP) to transport the fragile mRNA efficiently and safely into cells. Without the LNP technology to enable mRNA-based therapies, mRNA vaccines would not have taken a central role in the fight against COVID-19. It was fortunate the optimization of the delivery technology occurred in parallel with mRNA technology; although, for a completely different application, RNA interference (RNAi) induced gene silencing. In this review, we will focus on LNP-based delivery systems by covering the learnings obtained from siRNA delivery in pre-clinical studies to the eventual clinical experience of ONPATTRO^®^, which was the first RNAi based therapeutic product ever to be approved for human use. The success of siRNA-LNP therapeutics provided several valuable lessons applied in the development of mRNA-LNP currently evaluated in various applications ranging from prophylactic vaccines to gene editing. 

## 2. Lipid Based Delivery Systems

Alec Bangham’s work on biological membranes with the eventual description of liposomes in the early 1960s gave birth to a new field of drug delivery. This initiated decades of investigation that has brought forward multiple liposomal-based approved products addressing cancer therapy, fungal and viral infection, pain management, and photodynamic therapy [35]. The common theme among these products was the encapsulation of small molecules. These small molecules when delivered without a carrier were rapidly cleared, showed poor accumulation at target site, and/or were highly toxic. The encapsulation of these drugs improved the pharmacodynamic and pharmacokinetic properties by extending the circulation lifetime, controlling release, and increasing accumulation at sites of disease. Similar strategies were eventually realized and applied to nucleic acid drugs upon the discovery of cationic lipids. Although very active transfection agents in vitro [36], first generation permanently charged lipids, such as N-[1-(2,3-dioleyloxy)propyl]-N,N,N-trimethylammonium chloride (DOTMA), were toxic in vivo. Detergent-dialysis approaches were developed in the early to mid-1990s that used permanently charged cationic lipids such as dioleoyldimethylammonium chloride (DODAC) [37,38] and PEG-lipids [37,39,40] to improve control over particle formation and size and to generate more charge-balanced particles, thereby improving tolerability and enabling the delivery of nucleic acids in vivo. However, these first-generation delivery systems were non-optimal, with generally poor encapsulation and reliance on non-scalable processes. 

A fundamental shift in the formulation approach for nucleic acids, which became widely adopted and continues to the present as the ‘gold standard’ enabling excellent encapsulation and improved activity and safety in vivo, was developed at Inex Pharmaceuticals in the mid-1990s and described by Semple and colleagues in 1998 in the patent literature (WO/1998/051278), and later published [41,42]. In this approach, ionizable amino lipids, rather than permanently charged cationic lipids, were used to formulate nucleic acids at acidic pH (at which the amino lipids were positively charged) in the presence of ethanol. Upon removal of the ethanol and exchange of buffers, a charge neutral nanoparticle was formed with favorable physicochemical (e.g., diameters < 100 nm) and in vivo properties. 

To complement the breakthrough in efficient encapsulation of nucleic acids in LNPs, a scalable and robust manufacturing process was crucial. Different techniques such as sonication, co-solubilization with detergents followed by dialysis, or loading nucleic acids into preformed vesicles improved the encapsulation; however, it was the eventual combination of two critical processes, ethanol mixing [41,43] and controlled rapid mixing [44,45,46] when using the T-tube [47] or microfluidic devices [48], which enabled a robust, scalable, and reproducible formulation process to produce high level of nucleic acid encapsulation while maintaining a monodisperse size distribution. 

## 3. siRNA-LNP Development 

Work to encapsulate antisense oligonucleotides [41,49,50] and plasmids [37,38,39] provided critical learnings for the general encapsulation of nucleic acid molecules. However, successful transfection by plasmid delivery was hampered by the need for plasmid to be delivered first into the cell, and then subsequent migration into the nucleus where the DNA is transcribed prior to export of the corresponding transcript to the cytosol for translation. Although early work in plasmid showed some limited success, it was the application to siRNA that allowed the LNP technology to flourish since siRNA only required delivery to the cytosol where it is recognized and bound by the RNA-induced silencing complex (RISC) to induce silencing of target mRNA. In 2006, the first successful demonstration of systemically delivered siRNA using a LNP to down-regulate gene expression in non-human primates (NHP) was reported, knocking down serum apolipoprotein B (ApoB) levels [51]. This was the initial proof-of-concept showing the potential of LNP as a nucleic acid delivery vehicle in a higher species animal model to selectively regulate gene expression. 

The preclinical work by Alnylam Pharmaceuticals, AlCana Technologies (now Acuitas Therapeutics), and groups at University of British Columbia and Massachusetts Institute of Technology in the development of siRNA therapeutics showed that the ionizable lipid or lipid-like molecules along with the PEG lipid were critical determinants of the potency, safety, and stability of the LNP. It became evident that structural changes to the ionizable lipid design could significantly improve the siRNA activity, as demonstrated for the plasma clotting protein Factor VII decreasing ED_50_ (dose required to reduce target protein levels by 50%) from >10 mg/kg to 0.01 mg/kg to 0.005 mg/kg [52,53]. With hundreds of lipids synthesized and analyzed, a structure–activity relationship (SAR) was established showing the dependence of activity on the pKa of the lipid with the majority of lipids showing activity in gene silencing applications having a pKa ~6.2 to 6.5 [53,54]. Considering that the uptake mechanism of these LNP in the liver was mediated through a clathrin-mediated pathway [55], it was not surprising that the pKa of the ionizable lipid played an important role in activity by the destabilization of the endosomal membrane [56,57]. Endosomes are continuously acidified by the V-ATPase proton pumps [58], which transition the ionizable lipids that are neutral at physiological pH to a positive species once the endosomal pH drops below the ionizable lipid’s pKa. This charge would facilitate the interaction of the ionized lipid with anionic phospholipids in the endosomal membrane to adopt a non-bilayer structure, hexagonal (H(II)) phase leading to endosomal membrane disruption and release of payload [57]. 

Furthermore, the high efficiency of gene silencing in the liver with siRNA-LNP was also driven by the propensity of LNP to accumulate in the liver, as 60–70% of injected dose rapidly accumulated within 30 min of systemic administration [59]. This preferential accumulation in the liver was directed in large part by both the physiological structure of the organ as well as an endogenous targeting mechanism. LNP are structurally similar to chylomicrons, a lipoprotein particle composed of triglycerides, phospholipids, cholesterol, and proteins, and interact with circulating apolipoprotein E (ApoE) [60,61]. Using knockout models, Akinc and colleagues demonstrated that uptake of LNP by hepatocytes was largely dependent on both ApoE and the low-density lipoprotein receptor (LDL-R) [60,61]. However, not all delivery systems depend on ApoE targeting. In the case of the potent in vivo activity lipid-like molecule, C12-200, uptake was reported to be through an ApoE independent, micropinocytosis pathway [62,63,64]. 

## 4. Clinical siRNA-LNP Experiences

Following successful preclinical proof-of-concept demonstrations of the utility of LNP for the delivery of siRNA in monkeys [51], several first generation, systemically-administered LNP products entered clinical development in the late 2000s. In 2009, Alnylam Pharmaceuticals initiated a Phase 1 study of ALN-VSP, a dual siRNA product comprised of siRNAs directed against kinesin spindle protein (KSP) and vascular endothelial growth factor (VEGF) encapsulated in LNP, in patients with advanced liver cancers [65]. In addition, in 2009, Tekmira Pharmaceuticals initiated a Phase 1 study of PRO-040201, an LNP product containing an siRNA directed against ApoB in subjects with hypercholesterolemia. Although this program gave one of the first hints of siRNA activity in humans, achievable dose levels was ultimately hindered by the observation of immune stimulation and infusion-related reactions (IRRs) in some subjects. This was followed in 2010 with a Phase 1 study of another first generation LNP product, TKM-080301, containing an siRNA directed against polo-like kinase 1 (PLK1), in patients with advanced solid tumors. 

In addition to these, other first and second generation siRNA-LNP programs entered clinical trials through the early to mid-2010s (e.g., DCR-MYC for cancer, TKM-100201 and TKM-100802 for Ebola, and TKM-HBV for hepatitis B infection) [66], including in 2010, a first-generation LNP formulation known as ALN-TTR01, which entered Phase 1 clinical study in patients with transthyretin (TTR) mediated amyloidosis. At a dose level of 1 mg/kg, ALN-TTR01 resulted in a 38% reduction in transthyretin, compared with placebo [67]. Although these levels of TTR silencing were encouraging, parallel work to identify and characterize more potent amino lipids was ongoing. This resulted in the identification of what was to become the gold standard and clinically approved ionizable lipid (6Z,9Z,28Z,31Z)-heptatriacont-6,9,28,31-tetraene-19-yl 4-(dimethylamino)butanoate (DLinMC3DMA) [53]. Given the availability of a more potent lipid, along with a relatively high percentage of subjects (20.8%) experiencing mild-to-moderate IRRs in the ALN-TTR01 Phase 1 study, the ALN-TTR program evolved to a second-generation LNP containing DLinMC3DMA. In the Phase 1 study of ALN-TTR02, impressive reductions in TTR levels were observed at much lower doses, with peak mean reductions in TTR of 82.3 to 86.8% at ALN-TTR02 doses of 0.15 to 0.3 mg/kg, which were durable (56.6 to 67.1%) up to 28 days post-dose [67]. Interestingly, ALN-TTR02 also exhibited a lower incidence of IRRs compared to ALN-TTR01. Additional clinical validation of the activity of DLinMC3DMA was observed in a Phase 1 clinical trial of ALN-PCS02, containing an siRNA directed against proprotein convertase subtilisin/kexin type 9 (PCSK9), for the treatment of severe hypercholesterolemia. At intravenous dose levels ranging from 0.015 mg/kg to 0.4 mg/kg, circulating PCSK9 plasma protein and LDL-cholesterol were reduced (relative to baseline levels) by ~70% and ~40%, respectively, at 0.4 mg/kg [68]. 

ALN-TTR02 continued into pivotal clinical studies, including a Phase 3 multinational, randomized, double-blind, placebo-controlled study (APOLLO study) to evaluate efficacy and safety of ALN-TTR02 (patisiran) in TTR-mediated polyneuropathy (familial amyloidotic polyneuropathy-FAP), and was ultimately approved by the FDA in 2018, and in other jurisdictions shortly thereafter [69]. The approval of patisiran (ONPATTRO^®^) was a key milestone for both siRNA and LNP technology, providing clinical and regulatory validation of activity and safety of siRNA and LNP in a large multi-center study, and from an LNP perspective, establishing DLinMC3DMA LNP as the benchmark for future LNP-based drugs. Naturally, the clinical development success of siRNA-LNP led to increased interest in the potential for application of LNP to enable other nucleic acid modalities, particularly mRNA. 

## 5. mRNA-LNP Development 

In vivo delivery of mRNA with commercially available transfection reagents showed limited success [34]. Based on the success of siRNA-LNP and similarity of mRNA and siRNA both in terms of structure and site of action where delivery to the cytosol was sufficient for activity, it was hypothesized that the lessons learned from siRNA could be applied to mRNA. One of the first proof-of-concept applications of LNP with mRNA was shown by Pardi and colleagues in 2015 where an mRNA encoding the reporter gene firefly luciferase was successfully expressed in the liver and other tissues by various routes of administration with durable (up to 10 days) expression via intramuscular and intradermal administration [33] while Curevac in collaboration with Acuitas were first to demonstrate that similar LNPs were active in large animals [70]. 

**Figure 1:**
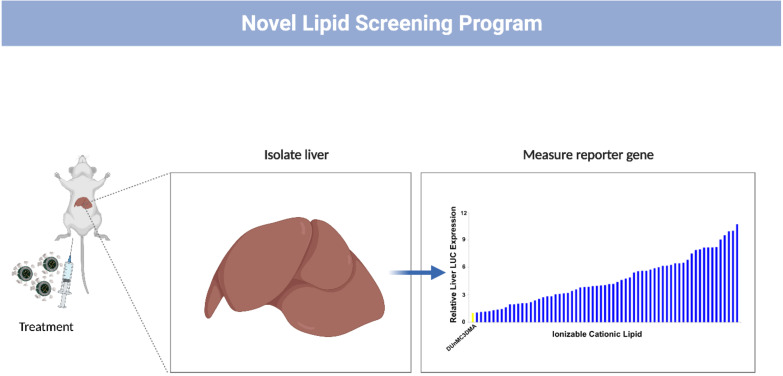
Novel lipid screening model. In vivo activity of novel ionizable lipids using a reporter gene firefly luciferase encoded in a mRNA are evaluated by intravenous administration in murine model.

Acuitas continues to dedicate significant efforts in the rational design of novel ionizable lipids with over 600 novel lipids designed and synthesized to date with a number outperforming (Figure 1) the clinically validated DLinMC3DMA used in the RNAi drug ONPATTRO^®^ [71,72,73,74,75,76,77]. Acuitas uses an iterative process correlating biophysical and structural features with potency and safety data to enable the rational design of lipids and LNP compositions with improved potency and therapeutic index [52,53,59,78]. The identification of novel ionizable lipids has enabled several partners and collaborators to investigate the performance of their respective nucleic acid payload [3,28,70,79,80,81,82,83,84,85,86,87,88,89]. Altogether, these efforts along with others [90,91,92,93,94] to improve the delivery platform have enabled proof-of-concept studies drawing significant success and excitement in several therapeutic applications including protein and enzyme replacement therapy, passive immunization, and prophylactic vaccines, of which several examples will be showcased below.

### 5.1. Protein/Enzyme Replacement Therapy

LNP delivery of mRNA encoding missing or non-functional proteins provides the ability to restore protein function in monogenic diseases. This can be applied in both protein and enzyme replacement therapies, which has garnered the interest in the potential to address different genetic disorders. As an example, Ramaswamy and colleagues demonstrated the therapeutic effect of delivering Factor IX using the Arcturus LUNAR LNP platform in a preclinical mouse hemophilia [95]. Phase Rx used a GalNAc-based delivery system to target the expression of ornithine transcarbamylase (OTC) mRNA in the liver of hyperammonemic murine model resulting in normalized plasma ammonia and urinary orotic acid levels, and prolonged survival [96]. Finally, Translate Bio’s delivery of alpha-galactosidase A mRNA with a LNP generated promising preclinical data in treatment of Fabry disease [97].

Moderna took a broader approach and targeted different genetic disease models including progressive familial intrahepactic cholestasis type 3 [98], citrin deficiency [99], hemophilia A [100], acute intermittent porphyria [101], Fabry disease [102], classic galactosemia [103], alpha 1-antitrypsin deficiency [104], and arginase deficiency [105]. From the several pre-clinical studies, Moderna advanced several programs into Phase 1 studies in 2019 through 2021, based on intravenous infusion of mRNA-LNP products, including mRNA-1944 (an LNP formulation of two mRNAs encoding the heavy and light chains of a human IgG antibody directed against Chikungunya virus) [106], mRNA-3927 (an LNP formulation of two mRNAs encoding the alpha and beta subunits of the mitochondrial enzyme propionyl-CoA carboxylase, a deficiency that leads to propionic acidemia characterized by recurring life-threatening metabolic decompensations and progressive multi-organ damage) [107], and mRNA-3705 (an LNP formulation of mRNA encoding methylmalonyl-CoA mutase, in which a deficiency leads to methylmalonic acidemia, a progressive and highly lethal disease characterized by episodes of metabolic decompensation) [108]. 

### 5.2. Passive Immunization

Generating an adaptive immune response against an infection generally takes weeks and often is too late to provide benefit to the afflicted. An alternative strategy to induce rapid protection is to provide patients with antibodies against the pathogen; however, designing and producing an effective antibody is often a long, tedious, and expensive process. These limitations could be potentially overcome through the use of mRNA-LNP encoding the heavy and light chains of antibodies. This approach was successfully demonstrated in different applications ranging from protection against pathogenic infection such as rabies, influenza B, and Chikungunya, to toxins such as botulinum toxin [80,106]. Similarly, with a more difficult infectious model, Pardi and colleagues designed mRNA encoding a broadly neutralizing antibody targeting HIV and showed successful protection of humanized mice upon HIV-1 challenge [31]. This strategy was also applied in cancer therapy with the delivery of mRNA encoding trastuzumab, Her-2 antibody [109] or rituximab, CD20 antibody [80], both respectively showing anti-cancer activity.

### 5.3. Prophylactic Vaccines

The largest and most advanced mRNA-LNP therapeutic has been in the area of prophylactic vaccine development. mRNA-LNP induced immunogenicity was demonstrated in Zika [32,110], Dengue [111], human cytomegalovirus [112], Ebola virus [113], and flavivirus [114] infections to name a few examples. In the next few sections, we will review recent proof-of-concept studies that are prime candidates for clinical development as prophylactic vaccines where the preventative measures can be improved to no current available vaccines. We will then complete this section by reviewing a real-life case study in the development of mRNA vaccine against SARS-CoV-2.

#### 5.3.1. Influenza

Seasonal influenza is an infectious disease that is easily transmissible resulting in 3–5 million cases with severe illness worldwide annually as reported by WHO (https://www.who.int/news-room/fact-sheets/detail/influenza-(seasonal) (accessed on 25 November 2021)). During the COVID-19 pandemic, the worldwide occurrence of influenza infection was dramatically reduced as a result of practices used to curb SARS-CoV-2 spread such as social distancing, mask implementation, and regular hand washing. With the expectation that influenza will again become a common infectious disease that returns yearly, as part of the annual practice, vaccines will continue to be an approach to stave away the severe symptoms and deaths caused by the different influenza strains, predominantly influenza A and B. According to CDC, the typical flu vaccines are designed as trivalent (two influenza A and one influenza B) or quadrivalent (two influenza A and two influenza B) options based on live attenuated virus, recombinant vaccine, or inactivated virus vaccine that are yearly updated (https://www.cdc.gov/flu/prevent/keyfacts.htm (accessed on 25 November 2021)). The efficacy of a flu vaccine fluctuates yearly as dominant influenza virus change through antigen shift and antigenic drift (reviewed in [115]). To address the ever-changing influenza virus, Geall and colleagues used self-amplifying (sa)RNA-LNP as proof-of-concept to rapidly develop an influenza vaccine for preclinical evaluation. This entailed identifying the sequence of the target virus, designing the saRNA to encode the antigens, encapsulation in LNP, and in vivo validation [116,117]. This work demonstrated protective levels of hemagglutinin inhibition (HI) titers based on the hemagglutinin (HA) antigen [117], while subsequent work explored a different antigen (NP and M1) showing both humoral and cellular responses [116]. This was recapitulated with the non-replicating mRNA modality when Moderna demonstrated mRNA-LNP-induced protection in preclinical animal challenges and immunogenicity (both humoral and cellular responses) in NHP with accompanying immunogenicity and safety readouts in human trials [118,119,120]. 

Due to the existence of various strains and the virus adapting and escaping from immune responses, current vaccines only have limited success in suppressing seasonal influenza with efficacy between 10 and 60% from 2004 to 2020 [115]. A solution to control and prevent seasonal influenza is to develop a universal influenza vaccine. With previous success in using mRNA-LNP to provide protection in Zika virus infection [32], Pardi and colleagues looked to develop a universal influenza vaccine by coding the full length influenza virus HA in a modified mRNA delivered by LNP, which induced both HA head and stalk specific antibodies [30]. Due to the relatively invariant nature of the stalk between different influenza strains, the presence of stalk specific antibodies resulted in the full protection in animals challenged with homologous and heterologous virus strains [30]. Similarly, broader protection was also realized by incorporating multiple antigens, which was achieved due to the modular nature of the mRNA-LNP platform. In a subsequent study, Freyn, Pardi and colleagues evaluated the immunogenicity and protection of mRNA-LNP vaccines consisting of mRNA encoding several different antigens of the virus (HA stalk, neuraminidase, matrix-2 ion channel, and nucleoprotein) with the objective to broaden the protective efficacy and redundancy [121]. It was observed that while each separate antigen provided limited protection, the combinatory approach provided protection against an array of influenza strains [121]. At doses of 0.05 µg per antigen, the protection of the combinatory regimen provided promising data to support the concept of an effective universal influenza vaccine. This could be improved by combining with a targeting LNP [122] or applied with the self-amplifying RNA, as direct comparison between mRNA and saRNA showed similar protection, however, achieved at much lower dose for saRNA [123]. 

#### 5.3.2. Malaria Vaccine

Malaria is a life-threatening infectious disease transmitted by female mosquitoes carrying a plasmodium parasite. According to WHO, 229 million cases were reported in 2019 with 409,000 deaths (https://www.who.int/news-room/fact-sheets/detail/malaria (accessed on 25 November 2021)). The major risk groups include infants and children under the age 5, which account for 67% of malaria deaths. Currently, preventions and treatments are available; however, drug resistance and waning protection towards malaria necessitates better alternatives. The only approved vaccine is RTS, S/AS01, which provides an initial ~70% vaccine efficacy that eventually wanes to 30–55% [124,125] while other vaccine candidates are to be evaluated in clinical trials [126,127,128,129]. Considering the efficacy of mRNA-LNP as prophylactic vaccines, Mallory and colleagues recently demonstrated the protection of mice in a rodent malaria challenge model after immunizing with mRNA encoding Plasmodium falciparum (Pf)CSP in LNP similar to those used in COMIRNATY^®^ inducing both humoral and cellular responses [82]. Moreover, immunizing NHP with a mRNA-LNP encoding a novel antigen PfGARP (glutamic acid-rich protein) provided partial protection from a challenge showing the anti PfGARP antibody attenuated parasite growth [130]. With promising but limited success, Raj and colleagues foresee a combinatory vaccine regimen to provide improved treatment against liver and erythrocyte infection. Both of these reports show promising alternative vaccines to RTS, S/AS01, which can be further refined and provide greater protection. Commitment to use the mRNA platform in delivering malaria vaccines has been announced by BioNTech (https://investors.biontech.de/news-releases/news-release-details/biontech-provides-update-plans-develop-sustainable-solutions (accessed on 25 November 2021)) with full support from the European Commission (https://ec.europa.eu/commission/presscorner/detail/en/speech_21_3864 (accessed on 25 November 2021)). 

#### 5.3.3. HIV Vaccine

As of 2020, 37.6 million people were currently infected with the human immunodeficiency virus (HIV) retrovirus (https://www.who.int/news-room/fact-sheets/detail/hiv-aids (accessed on 25 November 2021)). Over time, HIV positive patients eventually develop acquired immunodeficiency syndrome (AIDS) due to chronic and targeted infection of a variety of immune cells, which makes compromised individuals susceptible to diseases such as cancer and other infectious diseases. Pharmacological interventions are in place to reduce the viral load; however, preventative measures are not available. Various attempts to generate a successful HIV vaccine using traditional means had limited progress due to the high rate of genetic variability/mutability (reviewed in [131]). Although passive immunization using mRNA-LNP has shown success in protecting mice when HIV-challenged [31], an adaptive immune response would be preferred to confer a longer lasting protection. Using RNA-LNP as a platform to develop HIV vaccines, Blakney and colleagues demonstrated that saRNA-LNP formulations resulted in significant detection of HIV Env gp140 IgG in mice [132]. More significantly, Weissman, Kariko, Haynes, and colleagues used mRNA-LNP to generate a broadly neutralizing antibody with promising results in rabbits and NHP; however, the high degree of genetic variability of the HIV still prevented generation of a broad and durable neutralizing antibody after five injections [29]. Subsequent work with mRNA redesigned to encode soluble HIV-1 Env trimers with stabilizing mutations elicited neutralizing antibodies over the course of 41 weeks; however, the high level of titers fell ~10-fold from the last immunization [87]. Authors recognized that an effective vaccine will require further identification of additional HIV mutations that stabilize the HIV-1 envelope along with multivalent Env immunization for a successful mRNA-LNP vaccine [87] as multivalent strategy has proven to be effective [133]. Currently, various HIV vaccine development programs supported by the Bill and Melinda Gates Foundation are underway with Moderna targeted to initiate clinical trials with mRNA-1574 and mRNA-1644 (https://s29.q4cdn.com/745959723/files/doc_presentations/program-detail/other-vaccines/updated-pdfs/HIV-(11.04.21).pdf (accessed on 25 November 2021)).

#### 5.3.4. Herpes Simplex Virus

Herpes simplex virus (HSV) is a contagious infection caused by two subtypes, HSV-1 and -2, which is highlighted by blisters that become sores. In 2016, it was reported by the WHO that between 122 and 192 million people live with genital HSV-1 infection and 491 million people live with HSV-2 between the ages of 15–49 (https://www.who.int/news-room/fact-sheets/detail/herpes-simplex-virus (accessed on 25 November 2021)). HSV-2 is the main cause to genital herpes. Herpes can be alleviated with antiviral drugs such as Famvir, Zovirax, and Valtrex; however, there are no existing cures. With no approved HSV vaccine, studies led by Friedman compared the immunogenicity of protein-based vaccine adjuvanted with CpG/alum and mRNA-LNP encoding an entry glycoproteins gD and two additional immune evasion glycoproteins gC and gE [134,135]. The comparison showed that mRNA-vaccines provided an advantage with serum and vaginal IgG ELISA titers, neutralizing antibody titers against HSV-2 as well as HSV-1, antibodies that blocked evasion associated with gC and gE domains, cellular responses measured by CD4+ T cell responses, Tfh cell and GC B cell responses that ultimately protected the mice and guinea pigs from a HSV-2 challenge and genital lesions [134,135], which also provides protection for newborn pups infected intranasally with HSV-2 [136]. 

## 6. From Concept to Reality: Approval of the First mRNA-LNP Vaccine

The most active area of clinical development for mRNA-LNP technology, by far, has been in the area of mRNA vaccines. Clinical evaluation of mRNA-LNP vaccine platforms was well underway at the time that SARS-CoV-2 was being recognized as a potential pandemic. In January of 2020, CureVac reported interim safety and immunogenicity data from a Phase 1 study of CV7202, a novel prophylactic mRNA-LNP vaccine against rabies (using LNP provided by Acuitas). Two 1 or 2 μg doses of CV7202 were well tolerated and elicited rabies neutralizing antibody responses in all recipients that met WHO criteria for protection [137]. Similarly, Moderna was also highly active in the mRNA-LNP vaccine space for a number of infectious diseases. As a result, in early 2020 Moderna, BioNTech/Pfizer and CureVac were ready to rapidly initiate clinical mRNA vaccine programs to address SARS-CoV-2.

**Figure 2:**
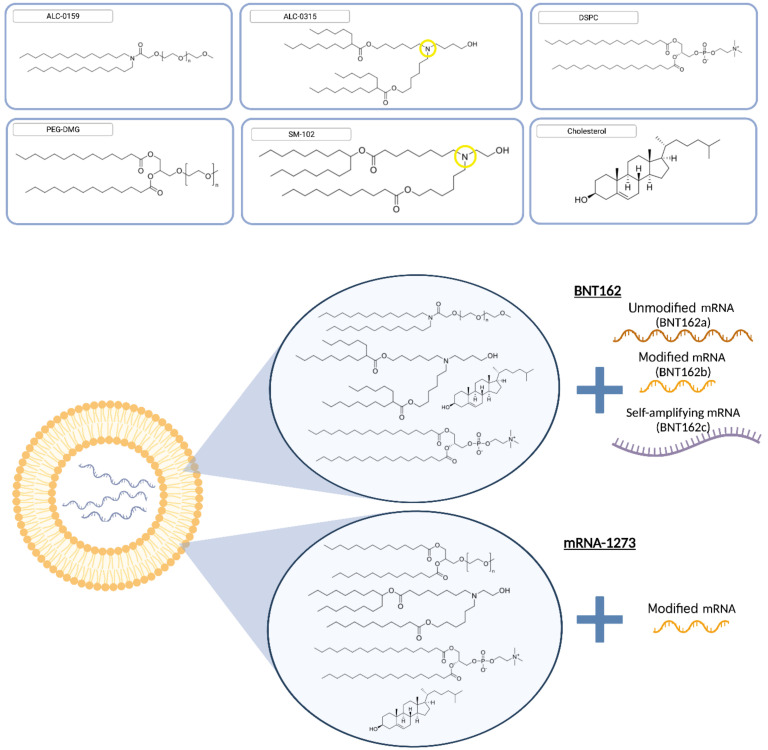
Lipid components in approved mRNA-LNP vaccines. BNT162b2 consisted of ALC-0315 ((4 hydroxybutyl)azanediyl)bis(hexane-6,1-diyl)bis(2-hexyldecanoate), ALC-0159 (2-[(polyethylene glycol)-2000]-N,N-ditetradecylacetamide) and two naturally-occurring lipids DSPC 1,2-distearoyl-sn-glycero-3-phosphocholine and cholesterol (https://www.ema.europa.eu/en/documents/assessment-report/comirnaty-epar-public-assessment-report_en.pdf (accessed on 25 November 2021)) while mRNA-1273 used SM102 9-Heptadecanyl 8-{(2-hydroxyethyl)[6-oxo-6-(undecyloxy)hexyl]amino}octanoate, PEG2000-DMG 1,2-dimyristoyl-rac-glycero-3-methoxypolyethylene glycol-2000, DSPC and cholesterol (https://www.ema.europa.eu/en/documents/assessment-report/spikevax-previously-covid-19-vaccine-moderna-epar-public-assessment-report_en.pdf (accessed on 25 November 2021)). The ionizable Nitrogen that drives the pKa of ALC-0315 and SM-102 are circled in yellow. BNT162 trials were based on three different modalities: unmodified mRNA (BNT162a), modified mRNA (BNT162b), and saRNA (BNT162c) while mRNA-1273 relied on modified mRNA.

In developing BNT162, multiple mRNA vaccine candidates were advanced into clinical studies in parallel [138,139], each containing a different mRNA, in recognition of the emerging worldwide pandemic and the critical need to expedite the identification of a safe and effective vaccine. BioNTech/Pfizer evaluated four different mRNA-LNP products consisting of different three RNA modalities: nucleoside-unmodified (BNT162a), nucleoside-modified (BNT162b), and self-amplifying (BNT162c) (Figure 2). Moderna, on the other hand, focused on the nucleoside-modified mRNA design. Preclinical studies on the modified mRNA modalities showed immunogenicity with humoral and cellular responses protecting against infection in both rodent and NHP models [139,140,141,142]. BioNTech/Pfizer eventually selected BNT162b2 due to enhanced tolerability while producing comparable immunogenicity in human [89]. Overall, BNT162b2 is a prophylactic vaccine developed to prevent COVID-19 caused by SARS-CoV-2 infection, using LNP technology developed by Acuitas. BNT162b2 is comprised of nucleoside-modified RNA (modRNA) that encodes the SARS-CoV-2 spike glycoprotein (S) antigen, encapsulated within a LNP formulation containing four lipids: ALC-0315, an ionizable amino lipid ((4 hydroxybutyl)azanediyl)bis(hexane-6,1-diyl)bis(2-hexyldecanoate), ALC-0159, a PEG-lipid (2-[(polyethylene glycol)-2000]-N,N-ditetradecylacetamide), and two naturally-occurring lipids (DSPC, 1,2-distearoyl-sn-glycero-3-phosphocholine and cholesterol) (Figure 2). It has been shown to be highly effective at preventing laboratory-confirmed COVID-19 illness in adults (95% protection) [3] and adolescents 12–15 years old (100% protection) [88] without evidence of previous infection with emergency use approval in children between 5 and 11 years at a reduced dosage. 

## 7. Lessons of Sourcing Material and Manufacturing Leading to Commercialization Scales

In late 2020, emergency use authorization was granted for BioNTech/Pfizer’s SARS-CoV-2 vaccine BNT162b2 (COMIRNATY^®^), making it the first mRNA therapeutic to receive regulatory authorization [3,88,89] followed soon after by Moderna’s mRNA-1273 (Spikevax^®^), validating the clinical potential of mRNA-based drugs and LNP technology, both less than one year from the onset of the global pandemic. To accomplish this, clinical trials were run concurrent to overall commercialization of the production processes, which is not the traditional approach for developing pharmaceutical drugs. This required significant financial commitments to secure raw materials, equipment, and production facilities prior to having clinical results to verify the vaccine was safe and effective (https://www.forbes.com/sites/nathanvardi/2020/05/20/the-man-betting-1-billion-that-pfizer-can-deliver-a-vaccine-by-this-fall/?sh=652bb233382e (accessed on 25 November 2021)).

**Figure 3:**
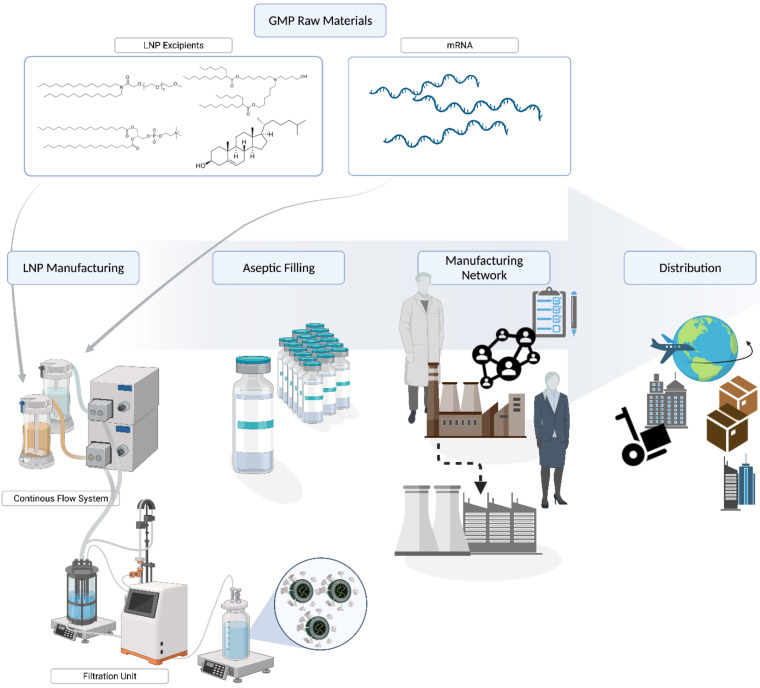
Manufacturing mRNA-LNP vaccines for COVID-19: From procurement of critical raw materials to LNP manufacturing and aseptic filling, expansion of manufacturing capacity, and distribution for administration.

The manufacture of mRNA-LNP is a specialized formulation process that combines the mRNA payload and four lipid components (Figure 2) under controlled mixing conditions to form LNP that are less than 100 nm in size. DSPC and cholesterol are commercially available pharmaceutical lipids, while the PEG and the cationic ionizable lipids are proprietary Acuitas lipids (WO2017075531A1 and WO2015199952A1). These require specific manufacturing expertise, equipment, and facilities to produce the pharmaceutical standards (Figure 3). Further, there are challenges in rapidly scaling up the production to ensure they are suitable for pharmaceutical applications. For example, the proprietary lipids were previously only required in gram quantities for clinical development. In contrast, the quantities of lipid excipients required to support the manufacture of the BioNTech/Pfizer vaccine to combat a global pandemic represented a momentous increase from previous scales. During clinical development, hundreds of grams of lipids are commonly required while commercial manufacture of a COVID-19 vaccine to deliver more than 2 billion doses would require hundreds of kilograms. Only specialized contract manufacturing organizations (CMOs) are registered to manufacture components for clinical and commercial products under the regulatory guidelines of good manufacturing practices (GMP), which ensures their quality and safety. The rapidity of the response to the current pandemic is owed in no small part to the coordination of GMP development by Acuitas of the key components as part of development of other LNP products prior to the appearance of SARS-CoV-2, and the capability of Avanti Polar Lipids to engage in further scale-up activities immediately to support late stage clinical and commercial supply to provide the springboard for the vaccine launch. Multiple CMOs and vendors for lipids such as Croda, AMRI, Evonik, and Merck KGaA and various raw materials have since been commissioned to support the manufacture of the BioNTech/Pfizer vaccine in order to meet the global need [143].

Another significant achievement was the development of a commercial manufacturing process for COMIRNATY^®^, which required substantial efforts from pharmaceutical development professionals across several organizations. As with the lipids, previous versions of LNP generation equipment were intended primarily to produce modest quantities (~1 to 10 g mRNA) of mRNA-LNP batches for clinical development programs, and although theoretically capable of at least 20–40 g mRNA output per hour, this magnitude in scale required for commercial vaccine production (i.e., 100s of grams mRNA per batch) had not yet been demonstrated with the equipment prototypes. In support of initial clinical development for the COVID-19 vaccine, Acuitas developed and transferred versions of the prototype manufacturing process to drug product CMOs such as Polymun Scientific to produce material suitable for clinical trials as a foundation for subsequent commercial scale-up development. Benefits of the Acuitas mixing system include a continuous process based on relatively simple equipment components that impart robustness characteristics favorable to large outputs and high flow rates, and was not prone to fouling as has been found in more complex systems [144,145]. The process is also amenable to increasing the output by scaling out the pilot scale mixing process via parallelization to generate equivalent drug product. 

The downstream purification process, Tangential Flow Ultrafiltration (TFF) also required significant scale up; unfortunately, this is a batch process and is limited by the capacity of the purification columns. TFF is commonly used for diafiltration and concentration in production of various pharmaceutical drugs. In this case, standard TFF processes had to be concurrently scaled up and optimized to ensure that the mRNA-LNP vaccine retained the desired product characteristics. This required design, procurement, and commissioning of specialized equipment suitable to manufacture commercial quantities of drug product during the early phase clinical development. Definitely, more elegant solutions could have been implemented given more time for product development; however, it is a testament to all involved that the core requirements were translated effectively to have production capabilities available to manufacture sufficient quantities of the vaccine to combat the pandemic. The COVID-19 pandemic marked the breakthrough of mRNA-LNP manufacturing technology. The worldwide demand could not have been foreseen a year ago and required an extraordinarily rapid expansion of manufacturing capacities for lipid and drug product manufacturing. The increase in demand required an immense scale up of the manufacturing processes by CMOs and the engagement of additional suppliers to meet global production needs. 

## 8. Next Generation COVID-19 mRNA-Vaccines

In addition to the development of COMIRNATY^®^ and Spikevax^®^, there were a number of RNA-based vaccines also submitted for clinical evaluation, which include programs at Sanofi in collaboration with Translate Bio (NCT04798027), GSK (NCT04758962), Providence Therapeutics in collaboration with Genevant (NCT04765436), Arcturus Therapeutics (NCT04668339), Imperial College of London (NCT04934111), and Curevac (2020-003998-22, NCT04652102). 

The design of the mRNA to encode an effective antigen that will generate broad neutralizing antibodies is critical. In one of the earlier clinical candidates, Robin Shattock’s group at Imperial College of London used saRNA-LNP to encode pre-fusion stabilized SARS-CoV-2 spike protein. In preclinical studies, they reported high levels (>10^6^ ng/mL titer) of SARS-CoV-2 specific IgG as well as a robust cellular response in mice [84]. This work led to the clinical trial COVAC1 (Study number: ISRCTN17072692) exploring doses from 0.1 to 10 µg (https://www.imperial.ac.uk/media/imperial-college/medicine/infectious-disease/COVAC1-Protocol-v8.0-15Dec2020.pdf (accessed on 25 November 2021)). Interim data suggested a dose response; although, 50% of seronegative participants did not seroconvert at 2.5 to 10 µg doses, which was hypothesized to be due to type I and III interferon production inhibiting translation and enhancing degradation of cellular mRNA. In a follow-up clinical trial that will be conducted in Uganda, the LNP-nCOV saRNA-02 was re-designed to dampen the type I and III IFN to increase antibody production. 

Meanwhile, work by Curevac showed promising preclinical readouts with generation of both humoral and robust T-cell responses as well as protection with viral challenge in rodent models with unmodified mRNA encoding full-length SARS-CoV-2 S protein with intact S1/S2 cleavage site and transmembrane domain, as well as K_986_P and V_987_P mutation (S-2P) [146], which was used in their clinical candidate CVnCoV. The Phase 1 safety and immunogenicity of CVnCoV candidate showed that doses from 2 to 12 µg were safe and well tolerated in healthy 18–60-year-old volunteers producing similar levels of neutralizing antibodies to convalescent sera when dosed at 12 µg [147,148]. The Phase 2b/3 study, the Herald study in 39,680 volunteers, conducted in Latin America and Europe, followed the suggested dose from the Phase 1 study, which consisted of dosing with a prime/boost regimen of 12 µg. Unfortunately and surprisingly, the ultimate readouts showed a lower vaccine efficacy of 48% in preventing disease of any severity [149] when compared to >94% reported in the Moderna and BioNTech/Pfizer trials [3,150]. Many elements such as timing and location of study where newer, more transmissible SARS-CoV-2 variants were dominant, mRNA modality, or lower doses were all potential contributing factors to lower efficacy. Although being very ambitious to target lower doses, an earlier NHP study indicated that the very low doses of 4 µg did not elicit an uniform nor robust immune response [151], which contrasted the success observed with the very low doses used in the earlier rabies vaccine clinical program [137,152]. A second-generation vaccine CV2CoV developed by Curevac in a joint program with GSK using a redesigned mRNA with optimized non-coding regions and enhanced antigen expression showed promising preclinical data by which the second-generation vaccine produced faster onset and >20 times the neutralizing antibody when compared to first-generation CVnCoV while inducing improved immunogenicity against ancestral and mutant strains [153,154]. Clinical trials are targeted to initiate at the end of 2021 (https://www.curevac.com/en/2021/08/16/second-generation-mrna-covid-19-vaccine-candidate-cv2cov-demonstrates-improved-immune-response-and-protection-in-preclinical-study (accessed on 25 November 2021)). 

## 9. Breakthrough and Challenges of mRNA-LNP

COMIRNATY^®^ became the first ever FDA approved mRNA-LNP product on the market for individuals older than 16 years of age. This approval will serve as a springboard for other mRNA-based treatments. The next application of mRNA-LNP to draw substantial excitement is gene editing. Extensive work with protein and enzyme replacement therapies as well as siRNA-based drug products have generated substantial interest in the correction of genetic diseases; however, these therapies are temporary and require chronic treatments to maintain the level of desired protein expression. To achieve a long-lasting effect, corrective gene editing by modifying DNA has been explored. The genome can be modified by inducing loss-of-function mutations or by gain-of-function by gene integration or gene correction [155]. The delivery of gene editing nucleases using viral vectors has shown varying levels of success; however, the inability to repeat dose due to adaptive host immunity towards the carrier, cytotoxicity, and potential off-target genomic integration have raised significant concerns. Exploration of non-viral gene editing as an alternative solution showed that the short-lived endonuclease expression mediated by mRNA-LNP still produced permanent gene editing. Encapsulating Zinc finger nucleases ZFN-mRNA in LNP were reported to enable >90% knockdown of TTR or PCSK9 gene [79] while the delivery of MEGA-TAL-mRNA also delivered with LNP resulted in up to 60% INDEL, which also corresponded to high levels (80%) of PCSK9 suppression and reduction in total cholesterol (Moore et 2018 ASGCT). 

In addition, CRISPR technology, for which the 2020 Nobel prize in Chemistry was awarded, -based studies using the codelivery of mRNA and guide (g)RNA in LNP were performed with promising outcomes in vitro and in vivo [156] particularly in cystic fibrosis models [157]. More recently, ground-breaking results by Intellia Therapeutics has generated significant excitement. Pre-clinical studies in murine models showed the successful editing of ~70% translating to reduction of >97% TTR serum protein for over 12 months with doses at 3 mg/kg when delivering optimized Cas9 mRNA and sgRNA in LNP [158], and when evaluated in NHP, a >95% reduction in serum TTR was achieved with a single administration (https://www.annualreports.com/HostedData/AnnualReports/PDF/NASDAQ_NTLA_2020.pdf (accessed on 25 November 2021). Intellia Therapeutics then followed by releasing interim data from a Phase 1 study of NTLA-2001, an in vivo gene-editing therapeutic agent, in patients with hereditary TTR amyloidosis with polyneuropathy (hATTR-PN). Following a single intravenous infusion, mean reductions (from baseline) in serum TTR after 28 days were 52% and 87% at dose levels of 0.1 mg/kg and 0.3 mg/kg, respectively [159]. The study is ongoing, with additional safety, durability of the response, and dose response data still to be collected; however, these initial results are promising and demonstrate initial proof-of-concept of therapeutic gene editing in humans. Unsurprisingly, when applied to base editors, using another CRISPR-based editing technology, it was possible to correct point mutations in mutant Fah in rodent models. Mutated Fah contributes to the fatal genetic disease hereditary tyrosinemia type I [160,161]. Furthermore, the activity of these base editors in large animals such as NHP was demonstrated in independent investigations by the Schwank group at University of Zurich and by Verve Therapeutics who disrupted the PCSK9 gene, leading to significant long-term reduction in LDL cholesterol [81,85], raising this approach as an exciting option for the treatment of heart disease.

Currently, the next breakthrough in the field of LNP is the ability to divert the nucleic acid payload away from the liver and allow access to extra-hepatic sites that would enable treatment of any genetic diseases from cancer to Alzheimer’s. The major issue to be addressed is the propensity of LNP accumulating in the liver upon systemic administration [59,60]. The current gold standard LNP based on DLinMC3DMA and ALC-0315 are lipids known to associate with ApoE, which are readily taken up by the liver. In order to escape the liver, the strategy whereby increasing PEG-lipid content to prevent the ApoE binding and deviate the endogenous targeting away from the liver has shown limited success in prolonging circulation and redistributing the LNP to extrahepatic sites [59,60]. A potential strategy is to use a targeting ligand to facilitate the uptake of LNP to specific organs [162,163]. Studies led by the Muzykantov laboratory have demonstrated the successful conjugation of antibodies targeting vascular adhesion molecule PECAM-1 or ICAM-1 and re-directing the LNP accumulation away from the liver to the lung [164,165] or VCAM-1 and targeting to the cerebral endothelium during brain edema [165]. A similar approach, using CD-4 antibody conjugated to the surface of the LNP, directed uptake into all T cells (naïve, central, memory, and effector) in both spleen and lymph enabling the possibility to conduct chimeric antigen receptor (CAR) T cell therapy in vivo [166]. Targeting ligands can re-distribute the LNP to extrahepatic sites; however, these examples are only demonstrated in small research scale settings. The current commercial manufacturing of mRNA-LNP is dependent on multiple steps that are not necessarily amenable to adding the targeting aspect. To ensure the manufacturing success of targeting LNP, two processes must be optimized: large scale production of the ligand, often a biological drug substance that is difficult and costly to manufacture, and the conjugation step to couple the ligand to the LNP. In addition to ligand-conjugated LNP, alternative means such as changes to the lipid species have shifted the tropism of LNP [167,168], which highlights the importance of using different approaches to identify extra-hepatic LNP. 

## 10. Conclusions

The mRNA-LNP platform has the potential to revolutionize treatment of disease. This was initiated by mRNA-LNP vaccines playing a central role in the prevention and protection against COVID-19. Although drug reactions have been reported, ongoing studies to identify the root causes would allow for further improvements of mRNA-LNP as a vaccine. The validation of mRNA-LNP as a viable alternative platform for vaccine development provides hope that several refractory infectious diseases may finally be addressed. mRNA-based vaccines are attractive as they allow ease and rapidity of antigen design for in vivo evaluation to optimize efficacy. Moreover, the manufacturing experience to fulfill global scale demands shows that mRNA-LNP vaccines can be efficiently and swiftly scaled up to resolve current infectious diseases and any future outbreaks. In addition, with the fact that mRNA-LNP has been realized in different applications, mRNA therapeutics have drawn significant interest and investment from large pharmaceutical companies allowing partnerships to be established or strengthened between the key mRNA technology companies with large pharmaceutical corporations. This is well exemplified by Sanofi Pasteur announcement in June 2021 with a commitment of EUR 400 million in establishing “mRNA Center of Excellence” (https://www.sanofi.com/en/media-room/press-releases/2021/2021-06-29-10-00-40-2254458 (accessed on 25 November 2021)). All the excitement surrounding mRNA-based therapeutics can certainly be accredited to Dr. Katalin Kariko.

## Data Availability

Not applicable.
